# Definitive chemoradiotherapy with paclitaxel for locally advanced esophageal squamous cell carcinoma in older patients (PARADISE-1): a phase I trial

**DOI:** 10.1186/s12885-024-12653-4

**Published:** 2024-07-19

**Authors:** Kenro Hirata, Kayo Yoshida, Chikatoshi Katada, Akinori Watanabe, Takahiro Tsushima, Toshifumi Yamaguchi, Sachiko Yamamoto, Hideki Ishikawa, Yasunori Sato, Chiyo K. Imamura, Yusuke Tanigawara, Yoshinori Ito, Ken Kato, Yuko Kitagawa, Yasuo Hamamoto

**Affiliations:** 1 Division of Gastroenterology and Hepatology, Department of Internal Medicine, Keio University School of Medicine, 35 Shinanomachi, Shinjuku-Ku, Tokyo, 160-8582 Japan; 2 Keio Cancer Center, Keio University School of Medicine, 35 Shinanomachi, Shinjuku-Ku, Tokyo, 160-8582 Japan; 3 Department of Radiology, Keio University School of Medicine, 35 Shinanomachi, Shinjuku-Ku, Tokyo, 160-8582 Japan; 4 Department of Gastroenterology, Kitasato University School of Medicine, 1-15-1 Kitasato, Minami, Sagamihara, Kanagawa 252-0374 Japan; 5 Division of Gastrointestinal Oncology, Shizuoka Cancer Center, 1007 , Nagaizumi-Cho, Sunto-Gun, Shizuoka, 411-8777 Japan; 6 Cancer Chemotherapy Center, Osaka Medical and Pharmaceutical University, 2-7, Daigaku-Cho, Takatsuki, Osaka 569-8686 Japan; 7 Department of Gastrointestinal Oncology, Osaka International Cancer Institute, 1-3-3Higashinari-Ku, NakamichiOsaka, 537-8511 Japan; 8 Graduate School of Medical Science, Kyoto Prefectural University of Medicine, 3-2-17-2F Imabashi, Chuo-Ku, Osaka, 541-0042 Japan; 9 Department of Preventive Medicine and Public Health, Keio University School of Medicine, 35 Shinanomachi, Shinjuku-Ku, Tokyo, 160-8582 Japan; 10 Advanced Cancer Translational Research Institute, Showa University, 1-5-8 Hatanodai, Shinagawa-Ku, Tokyo, 142-8555 Japan; 11 Laboratory of Pharmacometrics and Systems Pharmacology, Keio Frontier Research & Education Collaborative Square at Tonomachi, Keio University, 3-25-10 Tonomachi, Kawasaki-Ku, Kawasaki, Kanagawa 210-0821 Japan; 12 Department of Radiation Oncology, Showa University School of Medicine, 1-5-8 Hatanodai, Shinagawa-Ku, Tokyo, 142-8555 Japan; 13 Department of Head and Neck, Esophageal Medical Oncology, National Cancer Center Hospital, 5-1-1 Tsukiji, Chuo-Ku, Tokyo, 104-0045 Japan; 14 Department of Surgery, Keio University School of Medicine, 35 Shinanomachi, Shinjuku-Ku, Tokyo, 160-8582 Japan

**Keywords:** Aged, Chemoradiotherapy, Esophageal squamous cell carcinoma, Geriatric assessment, Vulnerable populations

## Abstract

**Background:**

In older patients, esophageal squamous cell carcinoma (ESCC) is difficult to treat using standard therapies, including surgery and cisplatin-based chemoradiotherapy. Paclitaxel (PTX) has radiosensitizing activity. We conducted a phase I trial of PTX combined with radiotherapy to establish a standard therapy for locally advanced ESCC in older patients.

**Methods:**

Enrollment was conducted at six centers in Japan from April 2016 to September 2019. The participants were aged ≥ 70 years, had locally advanced ESCC, and were intolerant to surgery or unwilling. A fixed 60-Gy radiation dose was administered in 30 fractions. PTX dosing levels started at 30 mg/m^2^ weekly for 6 weeks. Depending on the number of DLTs, the dose was set to be increased by 10 mg/m^2^ or switched to biweekly. A geriatric assessment was performed before treatment using the Geriatric-8 screening tool. The primary endpoint was dose-limiting toxicity (DLT).

**Results:**

We enrolled 24 patients (6 per group); DLT was observed in one (grade 4 hypokalemia), one (grade 3 aspiration), two (grade 3 radiodermatitis, grade 3 esophageal hemorrhage), and two (grade 3 anorexia, grade 5 pneumonitis) patients in the weekly PTX 30, 40, 50, and 60 mg/m^2^ groups, respectively. All adverse events, except death in the 60 mg/m^2^ group, showed reversible improvement, and the safety profile was considered acceptable. The 2-year survival and complete response rates were 40.0% and 54.2%, respectively. There was a significant difference in survival between favorable and unfavorable Geriatric-8 scores.

**Conclusions:**

The recommended PTX dose with concomitant radiation was determined to be 50 mg/m^2^ weekly. Phase II trials at this dose are underway.

## Background

The incidence of esophageal squamous cell carcinoma (ESCC) has been increasing and is highest in the 60–70-year age group. In Japan, 45% of these patients are aged > 70 years [1]. However, most clinical trials for ESCC have been conducted in patients with good performance status (PS) aged < 70 or < 75 years.

Currently, the standard treatment for locally advanced ESCC is neoadjuvant chemotherapy or chemoradiotherapy followed by surgical resection [2, 3] or definitive chemoradiotherapy, including cisplatin, if the patient cannot tolerate surgery or refuses to undergo surgery [4]. Marker et al. and Han et al. reported poor surgical outcomes in patients aged 70–80 years [5, 6], and Booka et al. reported no benefit of neoadjuvant chemotherapy in 75–80-year-old patients [7]. However, these studies included non-vulnerable older patients who could tolerate surgery. Many older patients are offered nonoperative treatment in clinical practice due to high surgical complication risk and severe medical comorbidities [8].

Chemoradiation and radiation alone are the most common nonoperative therapies. In Japan, JCOG0909 and JCOG0502 were clinical trials conducted on patients aged < 75 years with stage II/III and stage I ESCC, respectively [4, 9]. Both trials involved therapies comprising 5-fluorouracil, cisplatin, and radiation and are considered the standard of care for nonoperative treatment in Japan. There have been few prospective clinical trials of nonoperative treatment for patients aged > 75 years with locally advanced ESCC. A phase II study of radiotherapy with docetaxel in older patients was planned but prematurely closed because of slow accrual [10]. As previously reported, most institutions belonging to the Japan Esophageal Oncology Group agreed that clinical trials for older individuals are warranted and chose stage II/III (non-T4) ESCC as an important investigational target [11].

Several retrospective studies have been conducted on older patients; one involved ≥ 70-year-old patients who received chemoradiation or radiotherapy as initial treatment for stage II − III ESCC during 2000 − 2007 [12, 13]. They reported high toxicity discontinuation and low survival rates. Additionally, cisplatin therapy is not possible in patients with impaired renal function. Therefore, the only effective treatment is radiation alone with conservative irradiation, and limited efficacy must be tolerated. Consequently, there is a need to develop regimens that can be safely and continuously administered to older or vulnerable patients.

Paclitaxel (PTX) is a semi-synthetic cytotoxic drug derived from the precursor 10-deacetylbaccatin III extracted from the needle leaf of European yew (*Taxus baccata*). PTX acts on the structure and function of microtubules to generate abnormal microtubule bundles, which in turn interfere with chromosome migration and block cell division during the M phase of the cell cycle. PTX dose does not need to be reduced even in patients with impaired renal function because PTX is metabolized in the liver. In a phase II clinical trial of PTX for metastatic ESCC in Japan comprising 53 patients, the overall response rate was 44.2%, with four patients achieving complete response (CR) [14]. PTX has a radiosensitizing effect because it is synchronized with the G2/M phase, which is most sensitive to radiation [15–17]. Reckzeh et al. reported that the combination of PTX and radiation had a high CR rate of 29% in a phase I/II study of non-small cell lung cancer [18].

The present study aimed to demonstrate that chemoradiation with PTX, which can be administered to patients with impaired renal function, is safe and effective even in older or vulnerable patients and is useful as a standard treatment. Hence, we first designed and conducted the PARADISE-1 trial, a phase I trial, to determine the recommended dose (RD) and administration of PTX.

## Methods

### Objectives

This trial’s objective was to estimate the maximum tolerated dose (MTD) and dose-limiting toxicity (DLT) of PTX in combination with radiotherapy in older patients with locally advanced thoracic ESCC and to determine the RD of PTX. The primary endpoint was DLT incidence at each dose level. The secondary endpoints were safety, 2-year overall survival (OS), 2-year progression-free survival (PFS), CR rate, pharmacokinetics of total and unbound PTX, and correlation of efficacy with geriatric assessment tools.

### Study design

The PARADISE-1 trial was a dose-finding phase 1 study conducted in six centers in Japan. Definitions for older or vulnerable individuals were as follows: i) patients aged 70–80 years with an Eastern Cooperative Oncology Group (ECOG) PS of 0–1 and blood creatinine concentration of ≥ 1.5 mg/dL; ii) patients aged 70–80 years with an ECOG PS of 2; and iii) patients aged > 80 years with a PS of 0 or 1. Key inclusion criteria were 1) histological diagnosis of squamous cell carcinoma in the thoracic esophagus; 2) clinical stages IB, II, or III (excluding T4) based on the 7th edition of Union for International Cancer Control-TNM classification; 3) amenability to 60 Gy of radiotherapy; and 4) older or vulnerable patients based on the definition above. Key exclusion criteria were multiple active cancers, active infection, and severe pulmonary fibrosis or emphysema.

**Fig. 1:**
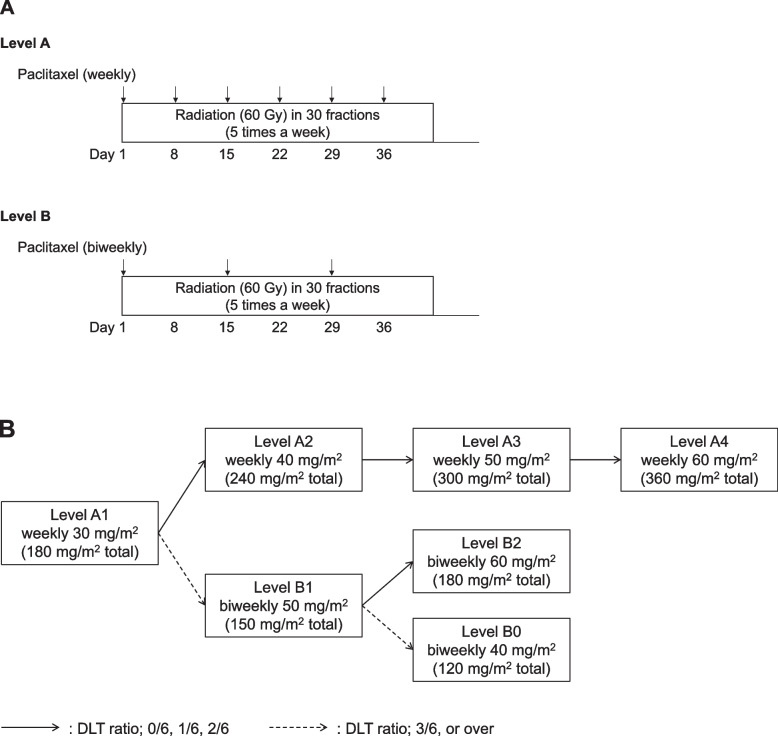
Study schema of the PARADISE-1 trial. **A** Protocol treatment at each level. **B** Study design. DLT, dose-limiting toxicity

Seven PTX doses were administered in this study: weekly (30, 40, 50, and 60 mg/m^2^: Level A1–A4) and biweekly (40, 50, and 60 mg/m^2^: Level B0–B2) (Fig. 1A and B). The number of patients enrolled at each level was set at six for safety reasons because the program targeted the older population. PTX dosing was started at Level A1. If fewer than two of the six patients in Level A1 had a DLT, the dose was increased to Level A2. If the cohort was shifted to Level A2, the cohort would not be shifted to Level B, which is a biweekly dose. Likewise, if there were two or fewer DLTs among the six patients enrolled in Level A2, dose escalation was repeated until a maximum Level A4 was achieved. During dose escalation at Level A, if there were three or more DLTs among the six patients enrolled, this level was considered MTD, and enrollment would be terminated. If PTX was increased to Level A4 and there were still two or fewer DLTs out of six enrolled patients, Level A4 was considered RD.

When there were three or more DLTs at Level A1, the cohort shifted to Level B1. If there were two or fewer DLTs at Level B1, the dose was increased to Level B2. Level B2 was RD when there were two or fewer DLTs and MTD when there were more than three DLTs. If there were more than three DLTs at Level B1, the dose was reduced to Level B0. If there were two or fewer DLTs at Level B0, Level B0 was designated as RD. Level B0 was the MTD if there were more than three DLTs. Dose reductions beyond level B0 were not to be conducted. In principle, the RD should be one level below the presumed MTD. Nonetheless, the final decision was made in consultation with the investigators, considering the toxicity of chemoradiotherapy and the drug’s relative dose intensity.

### Procedures

Patients received PTX intravenously for 1 h on days 1, 8, 15, 22, 29, and 36 in Level A and on days 1, 15, and 22 in Level B with concurrent 60 Gy of radiation in 30 fractions (Fig. 1A). In the A1, A2, A3, and A4 cohorts, patients received weekly doses of 30, 40, 50, and 60 mg/m^2^ of PTX, respectively, while in the B0, B1, and B2 cohorts, the patients received biweekly doses of 40, 50, and 60 mg/m^2^ of PTX, respectively (Fig. 1B). Radiotherapy was initiated on the same day as chemotherapy. Radiation was administered to the gross tumor and lymph nodes. Specifically, the primary esophageal tumor identified by endoscopy, computed tomography (CT), and optional positron emission tomography (PET)/CT imaging was contoured on the planning CT. Lymph nodes were considered positive if the short diameter was > 10 mm, or a round morphology suggested tumor involvement when the short diameter was 5–10 mm. Sufficient margins were generated to ensure adequate coverage. In this study, we chose not to include elective lymph node regions because of frailty in the patient population. All patients underwent three-dimensional conformal radiotherapy.

### Outcomes

The observation period for DLTs was from the start to 28 days after the end of chemoradiotherapy. The end of chemoradiotherapy was defined as the date of the last day of radiation therapy or the last day of PTX administration. DLT was defined as the following: i) grade 4 neutropenia persisting for > 4 days, even with granulocyte colony-stimulating factor treatment, ii) grade ≥ 3 febrile neutropenia lasting for > 4 days, iii) grade 4 thrombocytopenia, iv) clinically problematic grade ≥ 3 non-hematological toxicity, v) if PTX cannot be administered three consecutive times due to toxicity, vi) if a total of ≥ 14 days of radiotherapy suspension is required, and vii) discontinuation of protocol treatment for reasons other than patient refusal. In addition, clinically problematic grade ≥ 3 non-hematological toxicity corresponds to adverse events that make treatment continuation difficult. In other words, the following Grade 3 adverse events do not correspond to “non-hematological toxicity of Grade 3 or higher that is clinically relevant”: nausea, vomiting, anorexia, malaise, constipation, diarrhea, mucositis, hypersensitivity, transient abnormal test values for all metabolic/test items, and abnormal test values such as alkaline phosphatase derived from the underlying disease.

Tumor assessments using esophagogastroduodenoscopy (EGD) and CT scans of the neck, chest, abdomen, and pelvis were performed within 4 weeks before enrollment and 28–42 days after completion or discontinuation of the protocol treatment. Responses were assessed according to Response Evaluation Criteria in Solid Tumors (version 1.1) with the following three modifications: 1) Lymph node: short-diameter lesions measuring ≥ 10 mm were defined as lymph node lesions and evaluated as target lesions. For short-diameter lesions (5–9.9 mm), those clinically judged to be metastatic were considered non-target lesions, while those clinically diagnosed as non-metastatic were not considered lesions. A short diameter of < 5 mm was not considered a lesion; 2) primary lesion: added evaluation of the primary lesion by EGD. The primary lesion was evaluated as a non-target lesion. 3) Definition of CR: all lymph node lesions were < 5 mm in size. If there was a residual shadow of what was thought to be scar tissue on CT, a negative 2-[18F]-fluoro-2-deoxy-D-glucose-PET result was considered CR. Additionally, the primary lesion had to meet all of the following findings [19]: a) absence of endoscopic findings suggestive of neoplastic lesions. However, scarring, stenosis, iodine non-staining, and small granular prominences that were negative for cancer on biopsy were not considered “endoscopic findings suggestive of neoplastic lesions”; b) endoscopic biopsy of an area where the primary lesion was present before treatment was histopathologically free of cancer; c) the entire esophagus was observed on endoscopic examination; and d) no endoscopic findings (flat erosive changes, white moss) suggestive of active esophagitis. The severity of the adverse events was graded according to the National Cancer Institute Common Terminology Criteria for Adverse Events (version 4.0). OS was defined as the time from enrollment to death from any cause. PFS was defined as the time from enrollment to disease progression or death from any cause. The CR rate was defined as the percentage of patients who achieved CR with the full analysis set as the denominator. PTX pharmacokinetics were analyzed by collecting serum samples at the first PTX dose. This study employed three types of geriatric assessment tools: the Geriatric 8 (G8) screening tool score [20], the Instrumental Activities of Daily Living (IADL) score [21], and the Charlson comorbidity index (CCI) [22].

### Sample size calculation and statistical analysis

Figure 1B shows that this study’s minimum and maximum sample sizes were 12 and 24 participants at Levels A2 and A4, respectively. The enrollment period was set to 3.5 years, and the follow-up period was set to 6 months from the last patient’s enrollment. The time-to-event endpoints, including PFS and OS, were reported descriptively and estimated using the Kaplan–Meier method, and confidence intervals (CIs) were calculated using Greenwood’s formula. In the stratification analysis, hazard ratio (HR) and 95% CI were determined using the multivariate Cox proportional hazard model, and a log-rank test was used for comparisons between groups. All statistical analyses were performed using SPSS Statistics version 26 (SPSS Inc., Chicago, IL, USA).

### Pharmacokinetic sampling and assay

PTX pharmacokinetics were assessed for the first dose, and blood samples were obtained five times: before infusion, just before the end of a 1-h infusion, and at 10–60 min, 2–5 h, and 17–26 h after the infusion. Peripheral blood samples (5 mL) were drawn into vacuum tubes without anticoagulants and centrifuged at 3000 rpm for 10 min at room temperature. The resulting serum was frozen and stored at -80 °C until analysis.

The PTX concentration was determined using the ultra-performance liquid chromatography-tandem mass spectrometry method [23]. The unbound fraction of PTX in the serum, taken 10–60 min after the infusion, was obtained by equilibrium dialysis. The sample was prepared in a shaking incubator at 37 °C for 6 h using a 96-well microdialysis plate (HTD96b, HTDialysis, Gales Ferry, CT, USA), which was constructed of Teflon to minimize non-specific binding of the drug to the apparatus [24]. The dialysis compartments in each well were separated using a regenerated cellulose membrane (Dialysis Membrane Strips MWCO 12–14 kDa, HTDialysis). Experiments were conducted using 150-μL serum aliquots in an equal volume of Dulbecco’s phosphate-buffered saline.

Non-compartmental analysis using the Phoenix WinNonlin software (version 6.4; Certara, Princeton, NJ, USA) was performed to determine the area under the concentration–time curve (AUC) over time 0 (predose) to 24 h after dose administration (AUC_0-24_).

### Data management, control of data consistency, and quality control

The investigator or designated representative was required to fax all information required by the protocol after anonymization to protect patient privacy. The Kyoto Prefectural University of Medicine data center, independent of any hospital, checked patient eligibility, data completeness, validity, and consistency. The investigator or designated representative was obliged to provide clarification or respond to queries when generated. Additionally, each dataset was checked for errors or inconsistencies before merging with data from other sources or time points via the assigned study number to create a comprehensive dataset. Data access was limited to the authors and data center staff.

### Trial registration

This trial was registered in the University Hospital Medical Information Network Clinical Trials Registry (UMIN-CTR) (UMIN000020397, 29/12/2015) and the Japan Registry of Clinical Trials (jRCTs031180283, 15/3/2019).

## Results

### Study patients

From April 2016 to July 2019, 24 older patients with locally advanced ESCC were enrolled, six in each of the A1, A2, A3, and A4 cohorts. All patients were included in the efficacy analysis with a median follow-up period of 14.6 (Q1–Q3: 7.4–17.5) months.

### Patient characteristics

Table 1 shows the patient characteristics for each cohort. At enrollment, the median age of the patients was 83 (range: 73–92) years. Of the 24 patients (19 men and 5 women), only 25% had a PS score of 0. The most common primary site was the midthoracic region (54.2%), and the clinical stages were stage II (58.3%) and III (37.5%), with only one case of stage IB at Level A3. The values of the three geriatric assessment tools (G8, IADL, and CCI) assessed at enrollment did not differ significantly among the four cohorts. Additionally, patients’ results with the G8 screening tool showed greater variance than those of the IADL or CCI.

**Table 1:** Patient characteristics

	Level A1 (*n* = 6)	Level A2 (*n* = 6)	Level A3 (*n* = 6)	Level A4 (*n* = 6)	Total (*n* = 24)
**Median age, years (range)**	83.0 (80–87)	80.0 (73–87)	87.0 (80–92)	85.5 (83–89)	83.0 (73–92)
**Sex (male/female)**	4/2	6/0	3/3	6/0	19/5
**ECOG PS**					
0	1 (16.7%)	1 (16.7%)	2 (33.3%)	2 (33.3%)	6 (25.0%)
1	5 (83.3%)	3 (50.0%)	4 (66.7%)	4 (66.7%)	16 (66.7%)
2	0 (0.0%)	2 (33.3%)	0 (0.0%)	0 (0.0%)	2 (8.3%)
**Location**					
upper third	1 (16.7%)	1 (16.7%)	1 (16.7%)	0 (0.0%)	3 (12.5%)
middle third	3 (50.0%)	4 (66.7%)	4 (66.7%)	2 (33.3%)	13 (54.2%)
lower third	2 (33.3%)	1 (16.7%)	1 (16.7%)	4 (66.7%)	8 (33.3%)
**Clinical stage (UICC 7th)**					
IB	0 (0.0%)	0 (0.0%)	1 (16.7%)	0 (0.0%)	1 (4.2%)
II	4 (66.7%)	2 (33.3%)	4 (66.7%)	4 (66.7%)	14 (58.3%)
III	2 (33.3%)	4 (66.7%)	1 (16.7%)	2 (33.3%)	9 (37.5%)
**G8 screening tool score** (0–17)					
Mean ± SD (range)	11.2 ± 2.4 (8–14)	11.6 ± 2.6 (7–14)	10.5 ± 3.3 (6–14)	14.0 ± 1.4 (12–16)	11.8 ± 2.7 (6–16)
**IADL score** (male 0–5, female 0–8)					
male, mean ± SD (range)	5.0 ± 0.0 (5)	4.8 ± 0.4 (4–5)	4.7 ± 0.6 (4–5)	4.7 ± 0.5 (4–5)	4.8 ± 0.4 (4–5)
female, mean ± SD (range)	7.5 ± 0.7 (7–8)	-	7.7 ± 0.6 (7–8)	-	7.6 ± 0.5 (7–8)
**CCI** (0–37)					
Mean ± SD (range)	0.7 ± 0.8 (0–2)	1.2 ± 1.5 (0–4)	0.0 ± 0.0 (0)	0.2 ± 0.4 (0–1)	0.5 ± 0.9 (0–4)

*ECOG* Eastern Cooperative Oncology Group, *PS* Performance status, *UICC* Union for International Cancer Control, *G8* Geriatric 8, *SD* Standard deviation, *IADL* Instrumental activities of daily living, *CCI* Charlson comorbidity index

### Toxicity

Table 2 shows the DLTs and the best overall response. First, six patients were enrolled in Level A1; one was observed to have grade 4 hypokalemia, which was determined to be DLT. Next, according to the study design, six patients were enrolled in Level A2, and DLT of grade 3 aspiration was observed in one. Next, six patients were enrolled in Level A3; DLT occurred in two patients, grade 3 radiation dermatitis and grade 3 esophageal hemorrhage. Finally, six patients were enrolled in Level A4; DLT occurred in two patients, one with grade 3 anorexia and one with grade 5 pneumonitis, who died. Based on these results, the RD of PTX for radiotherapy was 60 mg/m^2^; however, after a comprehensive discussion among the investigators, it was decided that 50 mg/m^2^ would be the final RD.

**Table 2:** Dose-limiting toxicities, best overall response, and clinical pharmacokinetics in each cohort

	Level A1 (*n* = 6)	Level A2 (*n* = 6)	Level A3 (*n* = 6)	Level A4 (*n* = 6)	Total (*n* = 24)
**DLT events (n)**	grade 4 hypokalemia (1)	grade 3 aspiration (1)	grade 3 radiation dermatitis (1) and grade 3 esophageal hemorrhage (1)	grade 3 anorexia (1) and grade 5 pneumonitis (1)	
**Best overall response**					
Complete response (CR)	5	3	3	2	13 (54.2%)
Partial response (PR)	0	2	0	0	2 (8.3%)
Stable disease (SD)	0	0	0	1	1 (4.2%)
Progressive disease (PD)	0	0	0	0	0 (0.0%)
Non-CR/Non-PD	1	1	3	2	7 (29.2%)
Not evaluable or not assessed	0	0	0	1	1 (4.2%)
**Pharmacokinetics of PTX**					
AUC of total PTX (µM·h)	1.58–2.95	2.86–4.78^†^	3.46–6.09	4.37–5.99^†^	
AUC of unbound PTX (µM·h)	0.13–0.21	0.18–0.32^†^	0.21–0.32	0.27–0.36^†^	

*DLT* Dose-limiting toxicity, *PTX* Paclitaxel, *AUC* Area under the concentration–time curve

^†^*n* = 5

All 24 patients were included in the safety analysis (Table 3). The most common non-hematological adverse event was esophagitis, occurring in 91.7% of patients in all grades and 20.8% in grades ≥ 3. Anorexia was also present in 83.3% of the patients, with 12.5% having a grade ≥ 3. Hematological toxicities included anemia in all patients and neutropenia in 62.5% of the patients. Febrile neutropenia was observed in one patient at Level A3 but not at Levels A1, A2, and A4. No late effects that could be attributed to radiotherapy were reported during the observation period. This trial’s only treatment-related death was due to pneumonitis in an 83-year-old man with cT3N3M0 midthoracic ESCC. The patient was included in the Level A4 cohort, and a mild sore throat appeared 28 days after commencing the protocol treatment with PTX 60 mg/m^2^ and radiotherapy 60 Gy. The patient was admitted to the hospital 41 days after starting the protocol treatment because of difficulty in food intake and was diagnosed with radiation esophagitis. Symptoms of shortness of breath appeared 46 days after commencing the treatment protocol. A chest CT scan the following day revealed interstitial pneumonia, for which steroid pulse therapy was started (methylprednisolone 1000 mg/day). Diffuse pneumonitis was observed in both lungs, characterized as radiographically distinct from radiation pneumonitis, and was diagnosed as an adverse event of PTX. The patient was unresponsive to steroid treatment and died 60 days after the treatment commenced.

**Table 3:** Treatment-related adverse events

	Level A1 (*n* = 6)		Level A2 (*n* = 6)		Level A3 (*n* = 6)		Level A4 (*n* = 6)		Total (*n* = 24)	
	**All**	** ≥ Grade 3**	**All**	** ≥ Grade 3**	**All**	** ≥ Grade 3**	**All**	** ≥ Grade 3**	**All**	** ≥ Grade 3**
**Non-hematological adverse events**										
Esophagitis	5	1	6	1	5	1	6	2	22 (91.7)	5 (20.8)
Anorexia	6	1	4	0	4	1	6	1	20 (83.3)	3 (12.5)
Dysphagia	1	0	2	1	2	0	3	0	8 (33.3)	1 (4.2)
Nausea	1	0	1	0	2	0	2	0	6 (25.0)	0 (0.0)
Pneumonitis	0	0	1	0	0	0	3	2	4 (16.7)	2 (8.3)
Vomiting	2	0	0	0	0	0	2	0	4 (16.7)	0 (0.0)
Dermatitis radiation	0	0	2	0	1	1	0	0	3 (12.5)	1 (4.2)
Febrile neutropenia	0	0	0	0	1	1	0	0	1 (4.2)	1 (4.2)
Diarrhea	1	0	0	0	0	0	0	0	1 (4.2)	0 (0.0)
Oral mucositis	0	0	0	0	0	0	1	0	1 (4.2)	0 (0.0)
**Hematological adverse events**										
Anemia	6	2	6	0	6	2	6	1	24 (100.0)	5 (20.8)
Leucopenia	3	0	5	1	6	3	6	2	20 (83.3)	6 (15.0)
Neutropenia	1	0	5	0	5	2	4	0	15 (62.5)	2 (8.3)
Thrombocytopenia	2	0	2	0	1	0	2	0	7 (29.2)	0 (0.0)

### Pharmacokinetics of PTX

**Fig. 2:**
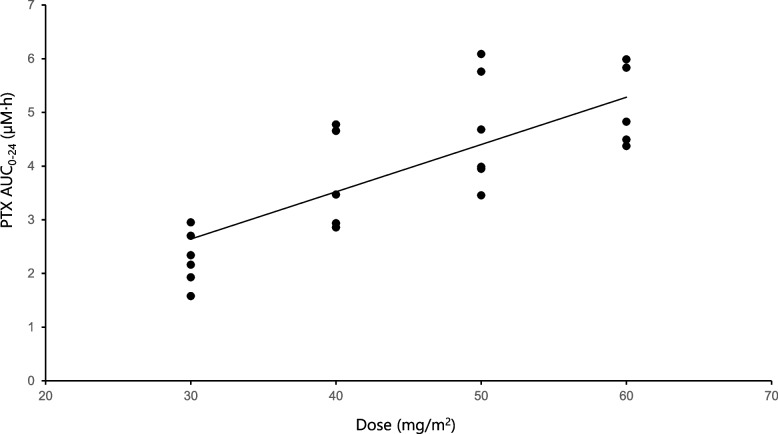
Relationship between dose and AUC_0-24_

PTX pharmacokinetics were measured in 22 of 24 patients (Level A1 and A3, six patients each; Level A2 and A4, five patients each). Table 2 shows the AUC_0-24_ of total PTX and unbound PTX. Linear pharmacokinetics of total PTX was observed within the dose range of 30–60 mg/m^2^ (Fig. 2).

### Efficacy

**Fig. 3:**
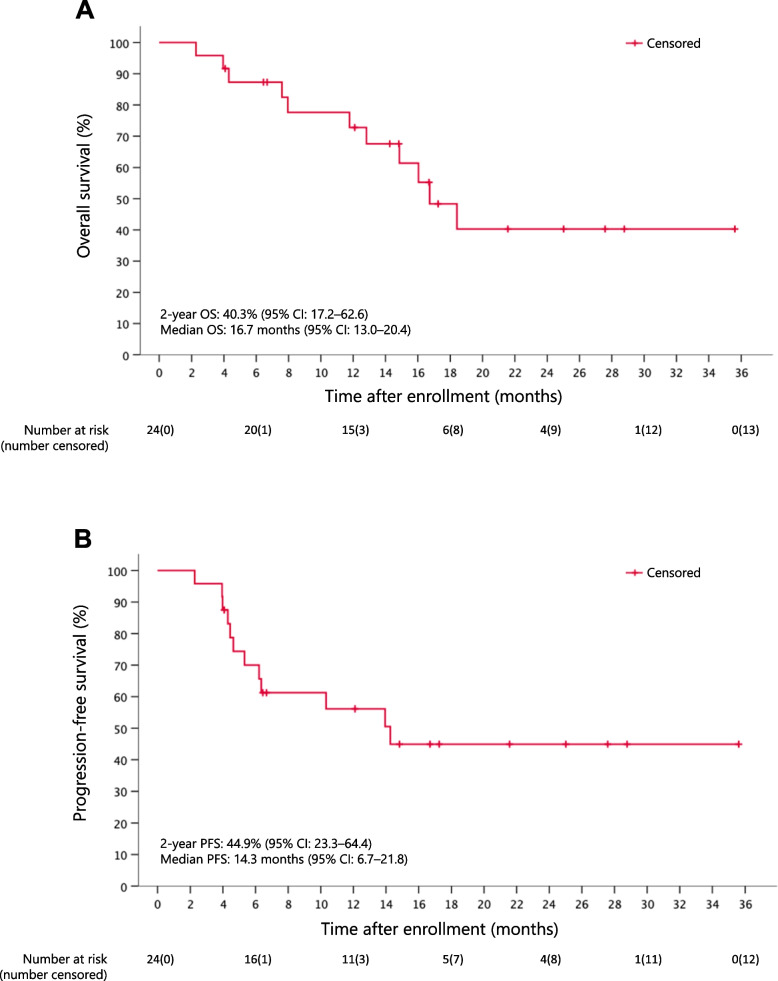
Kaplan–Meier plots of overall survival (**A**) and progression-free survival (**B**)

**Fig. 4:**
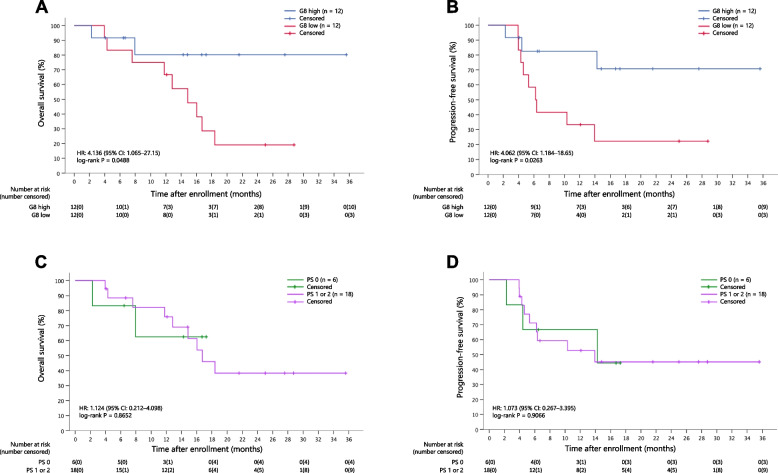
Kaplan–Meier plots of survival outcome based on G8 score and PS. **A** Overall survival by G8 score. **B** Progression-free survival based on G8 score. **C** Overall survival based on PS. **D** Progression-free survival based on PS. G8, Geriatric 8 screening

The rates of best overall response for the entire cohort were 54.2% (95% CI: 32.8–74.5) for CR, 8.3% (95% CI: 1.05–27.0) for partial response, 4.2% (95% CI: 0.11–21.1) for stable disease, 0% (95% CI: 0.00–14.2) for progressive disease, and 29.2% (95% CI: 12.6–51.1) for non-CR/non-progressive disease. One patient was not evaluated because he died of pneumonitis. By the data cutoff (January 16, 2020), 11 (45.8%) of the 24 patients had died. Follow-up patients in this study showed an estimated 2-year OS rate of 40.3% (95% CI: 17.2–62.6) and a median OS of 16.7 months (95% CI: 13.0–20.4) (Fig. 3A). Additionally, the estimated 2-year PFS rate was 44.9% (95% CI: 23.3–64.4), and the median PFS was 14.3 months (95% CI: 6.7–21.8) (Fig. 3B). Using a median G8 screening tool score of 12.5 as a cutoff point for OS, there was a statistically significant increase in OS in the high G8 group compared to that in the low G8 group (HR: 4.136; 95% CI: 1.065–27.15; log-rank *P* = 0.0488) (Fig. 4A). A statistically significant increase occurred in PFS (HR: 4.062; 95% CI: 1.184–18.65; log-rank *P* = 0.0263) (Fig. 4B). In contrast, when OS and PFS were compared in cohorts with PS of 0 and 1–2, there were no significant differences in OS (HR: 1.124; 95% CI: 0.212–4.098; log-rank *P* = 0.8652) and PFS (HR: 1.073; 95% CI: 0.267–3.395; log-rank *P* = 0.9066) (Fig. 4C and D).

## Discussion

Treatment choice should be individualized based on chronological age, PS, comorbidity, concomitant use of multiple drugs, and multidisciplinary geriatric assessment. Moreover, classifying older patients into those who should receive the same treatment as the young and those who should not is necessary. Additionally, it is essential to pursue treatment strategies for unfit patients by further dividing them into two groups: those who can be treated with aggressive treatment and those who are best suited for supportive care. The current study included older patients and those with impaired renal function who could not safely use cisplatin.

This study determined the RD and administration of PTX in combination with radiation as 50 mg/m^2^ once a week. In the patient group receiving 60 mg/m^2^ of weekly PTX, one grade 3 anorexia and esophagitis and one grade 5 event due to interstitial pneumonia were observed. Although the number of DLTs was two, it was less than the predefined three required to lower the dose; therefore, the investigators discussed and agreed that 60 mg/m^2^ of PTX weekly was unacceptable as deaths were observed. They concluded that a CR rate of 50% was obtained even at a dose of 50 mg/m^2^ and was also expected to be sufficiently effective, and decided on 50 mg/m^2^ as the RD instead of 60 mg/m^2^.

Several retrospective studies have reported 2-year OS rates ranging from 26.0 to 35.5% and median OS intervals ranging from 8.6 to 15.2 months in older patients treated with standard chemoradiation with fluorouracil and cisplatin [25–28]. Our study showed a 2-year survival rate of 40.3% and a median survival time of 16.7 months, suggesting that radiotherapy with PTX may be more effective. Recently, Yongling et al. reported the efficacy of chemoradiotherapy with S-1 in older Chinese patients with esophageal cancer [29]; however, S-1 cannot be used in patients with impaired renal function. The 2-year survival rate in their study was good at 53.2%. However, in our study, the participants’ median age was 77 years, younger than 83 years, and patients with impaired renal function were not included. In comparison, our study investigated chemoradiotherapy with PTX, which can be administered even to patients with impaired renal function, for those considered intolerant to standard chemoradiotherapy, including cisplatin. Although our study included even more vulnerable patients than the Chinese study, it demonstrated certain efficacy and safety.

To our knowledge, this is the first study to perform a geriatric assessment of ESCC prospectively. To assess the patients’ conditions, we used three different screening tools for geriatric assessment: G8, IADL, and CCI; however, the low variance of the values for IADL and CCI made them useless for patient assessment in our study. Contrastingly, the G8 score showed a large variance; when OS and PFS were evaluated by dichotomizing the median, a statistically significant prolongation of survival was observed in the group with a favorable G8 score. G8 has also been reported to be useful as a prognostic tool in lung [30] and head and neck cancer [31]. In treating older patients, some patients have a good ECOG PS but a short survival time [32]. In the current study, survival curves by ECOG PS showed no clear difference, suggesting that the G8 screening tool may be more useful than ECOG PS for predicting survival in patients with ESCC. The G8 screening tool can be considered for treatment selection and decision-making in geriatric cancer patients.

Although non-linear pharmacokinetics were reported for a 3-h infusion of PTX at 135‒225 mg/m^2^ [33], linear pharmacokinetics were observed for a 1-h infusion at 30‒60 mg/m^2^ in this study. This suggests that PTX metabolism is not saturated within 30‒60 mg/m^2^. For drugs highly bound to serum proteins, unbound drug exposure correlates better with toxicity and efficacy than total exposure because unbound drug molecules are more likely to traverse cell membranes and distribute into tissues to bind their targets. However, DLTs were attributed to radiation rather than PTX. Therefore, the relationship between drug exposure and toxicity could not be evaluated.

A limitation of this study is that 24 patients with different doses of PTX were considered together in the survival analysis and geriatric assessment. Additionally, the definition of older or vulnerable patients was determined by inter-investigator discussion from a clinical perspective as a group of patients who would be hesitant to receive cisplatin. Another limitation is that the final cause of death was not determined for all patients, even though the study was conducted in older and vulnerable patients who were also more likely to die from other causes. Furthermore, patient recruitment was difficult because the study was conducted in a rare population; hence, it took 3 years and 4 months to accumulate 24 patients.

## Conclusions

The results of this phase I study established an RD of 50 mg/m^2^ PTX in combination with 60 Gy/30 Fr radiation for older patients with ESCC who are considered refractory or intolerant to standard therapy. In addition, the G8 score may be a useful prognostic tool in older patients with ESCC. Furthermore, the phase II study (PARADISE-2) was initiated at 12 centers in Japan in October 2019 to evaluate the efficacy and safety of this regimen, with results to be reported at a later date.

## Data Availability

All data generated and analyzed during this study are included in this article.

## Abbreviations

AUC: Area under the concentration–time curve
CCI: Charlson comorbidity index
Cis: Confidence intervals
CR: Complete response
CT: Computed tomography
DLT: Dose-limiting toxicity
ECOG: Eastern Cooperative Oncology Group
EGD: Esophagogastroduodenoscopy
ESCC: Esophageal squamous cell carcinoma
HR: Hazard ratio
IADL: Instrumental Activities of Daily Living
MTD: Maximum tolerated dose
OS: Overall survival
PET: Positron emission tomography
PFD: Progression-free survival
PS: Performance status
PTX: Paclitaxel
RD: Recommended dose
